# What are the Clinical and Social Outcomes of Integrated Care for Older People? A Qualitative Systematic Review

**DOI:** 10.5334/ijic.6469

**Published:** 2022-09-07

**Authors:** Sara Karacsony, Helga Merl, Jane O’Brien, Hazel Maxwell, Sharon Andrews, Melanie Greenwood, Maryam Rouhi, Damhnat McCann, Christine Stirling

**Affiliations:** 1 University of Tasmania, Australia

**Keywords:** integrated care, older people, outcomes, health care systems, meta-aggregation

## Abstract

**Introduction:**

Older people with multiple chronic conditions have most to gain from successful integrated care models but there is a need to understand current evidence of outcomes for older people.

**Methods:**

A qualitative meta-aggregation method was used for the review. Systematic searching of CINAHL, PubMed (Medline), Web of Science, PsycINFO, Scopus and Cochrane identified an initial 93 papers, of which 27 were reviewed. Studies were selected according to the pre-defined protocol and quality assessed using The Joanna Briggs Institute Critical Appraisal Tools (JBIQARI). Eleven, peer-reviewed, English-language papers published between 2000 to 2020 were included.

**Results:**

Thirty-three findings were extracted and aggregated into six categories. Three synthesised statements were identified denoting outcomes of integrated care for older people. These indicate social participation and connectedness for older people and their families; the older person feeling motivated to engage in health goals when their preferences were taken into consideration; and older people experiencing support and wellbeing when a therapeutic relationship with a key worker is established.

**Discussion and conclusion:**

There was scant evidence of the older person’s voice within included studies and a limited focus on outcomes. Stronger evidence is needed to provide meaningful and robust evaluation of outcomes within integrated care models for the older person.

## Introduction

The concept of integrated care has emerged to address the growing chronic condition burden of global ageing and associated costs of care and delivery implications [1,2]. There is an expectation that moving away from the biomedical model of disease and symptom management to more social and integrated person-centred care models will improve indicators related to wellbeing and healthy ageing [3] and reduce health and social care costs [4].

Integrated aged care is expected to decrease fragmentation by connecting health and social care systems, organisations and services to provide a seamless, continuum of care built around the older person’s expressed needs [2]. Much literature concentrates on the integration of various health services, but this review focuses on the integration of health and social care (assistance that provides practical and personal support for people to age in place) using the following definition by Nolte and Pitchforth (2014, p.6).

“Initiatives seeking to improve outcomes for those with (complex) chronic health problems and needs by overcoming issues of fragmentation through linkage or coordination of services of different providers along the continuum of care.”

Integrated care is often seen as particularly important for older people, who have the greatest number of chronic conditions and comorbidities of any age group [5]. In Australia, between 35 and 60 percent of older Australians have two or more chronic conditions [6,7], with many living in the community with complex disorders such as dementia, sarcopenia and frailty [8]. These require holistic health and social care interventions to enable healthy ageing, wellbeing and quality of life whilst preventing further disability and avoidable admission to acute and long-term care facilities [8,9].

While integrated care models have traditionally had a clear focus on improving health outcomes, social care outcomes have been comparatively overlooked. Social engagement is a known contributor to functional independence and can prevent social frailty cascading into physical and cognitive frailty [10]. Social care services can avert premature transition to residential care [11] or avoidable admission to hospital care [12,13]. Unfortunately, there are few examples of integrated social and health care services [4].

At the micro or individual level, integrated care seeks to improve the quality of care for individual patients, service users and carers by ensuring all services are well coordinated around their needs [14]. Important enablers to the older person’s experience of integrated care have been identified as access, information, communication, and coordination, including referrals and care transitions [15]. Low levels of health literacy, particularly for older persons, is a known weak spot in the operationalisation of these enablers [16]. The effects of integrated care are perceived to improve quality of care, increase patient satisfaction and improve access to care, although there is little reported information of outcomes for service uses [17].

While the shift to integrated care was heralded as a panacea to the current fragmented and costly aged care system, it remains unclear whether integrated care has taken place and achieved its goals at the micro level or level of the individual. It is now imperative to examine whether integrated care initiatives are meeting the health and social care needs of older people and what outcomes are being reported. This review addresses the question: “What are the clinical and social outcomes of integrated care for older people, their families and carers?”

## Method

To address the aim, a systematic qualitative meta-aggregation review was conducted by the team following the Joanna Briggs process [18]. The meta-aggregation process is underpinned by pragmatism and aims to generate a set of statements linked in a transparent way to the data to produce practical ‘lines of action’ and detailed, measurable and specific recommendations for practitioners and policy makers [18]. This review has been registered with Prospero CRD42020192345.

### Search strategy

This review considered studies that contained qualitative data from any methodology and/or research design and included mixed methods studies with qualitative data as primary data source. We excluded all studies that report quantitative data including mixed methods studies when quantitative data was the primary data source.

Peer-reviewed articles in English from January 2000 to September 2020 were selected. The inclusion criteria were formulated according to the PICO format (Participant, Interest, Context, Outcomes). ‘Participant’ was defined as persons aged 65 years or over or 50 and older if focused on indigenous populations and includes families’ and carers’ perspectives. ‘Interest’ was defined as integrated care at the micro level. ‘Context’ was all settings and eligible population types, or groupings included within the review. Studies captured outcomes related to exercise, cognitive stimulation, and community engagement, for example, as these are all important aspects of healthy ageing and can be found in programs such as reablement programs, and those that have social prescribing as an intervention. This systematic review considered the evidence base using the definition of integrated care by Nolte and Pitchforth (2014) that seeks to improve outcomes for those with complex chronic health problems.

Two researchers (SA, HM) identified the following search terms: Integrated care OR Coordinated care OR Reablement OR Interdisciplinary care OR Integrated service delivery OR Patient-centred OR Social prescribing AND Quality of life OR Health-related quality of life OR HRQOL OR Wellbeing OR Functional OR Social OR psychosocial AND Older people OR Geriatric OR Elderly OR Senior OR 65 years AND Qualitative study OR Mixed-method OR Qualitative Research OR mixed methods study. The use of Boolean Operators was used to access studies.

**Figure 1:**
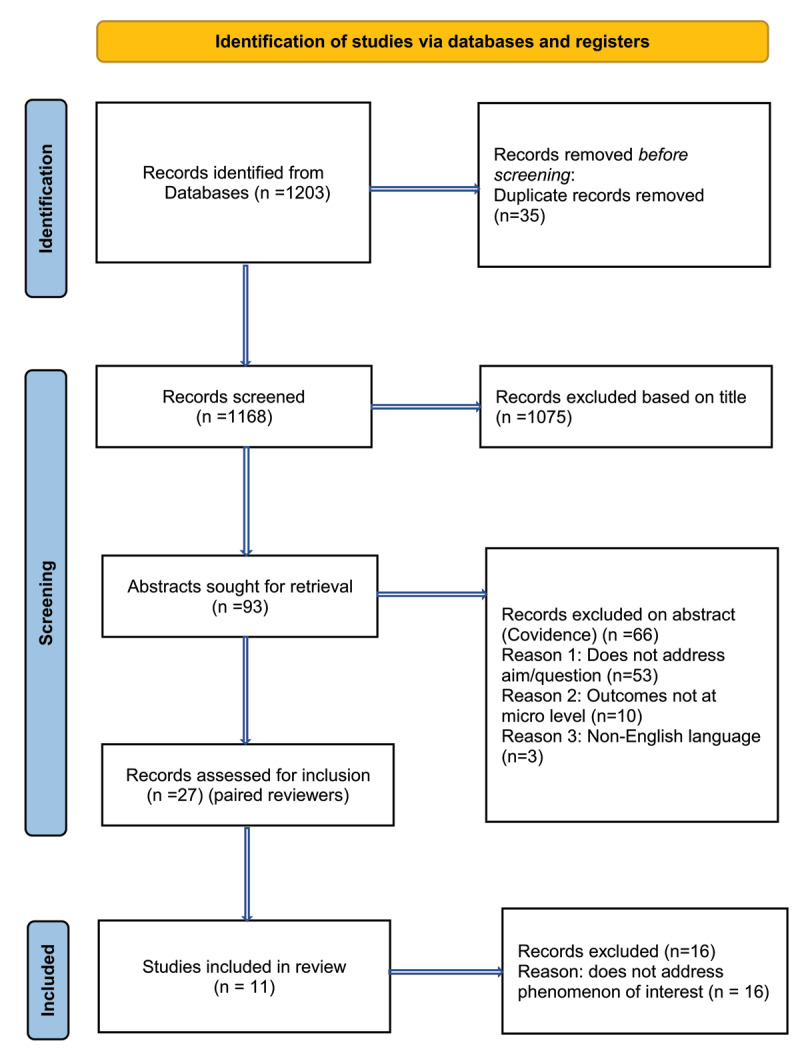
PRISMA flow diagram showing the study selection process.

MR then searched for English language papers on the following electronic databases in consultation with a subject relevant librarian: CINAHL, PubMed (Medline), Web of Science, PsychINFO, Scopus, Cochrane. The search method identified 1203 papers. All identified citations were collated and uploaded to an EndNote library. Duplicates (n = 35) were manually removed prior to selection of studies (Figure 1) by (MR). Titles and abstracts of each citation were then screened against the inclusion criteria by two independent reviewers who were paired for this activity (paired reviewers: CS-HM; MG-SA, SK-HM, KB-PM, JO’B-DM). Studies that met the inclusion criteria were retrieved in full and their citation details imported into the Covidence data extraction tool (licence number 73933), a web-based software platform to assist extraction data for systematic and other research reviews that require screening citations and full text, assessing risk of bias, or extracting study characteristics and outcomes [19]. A full text copy of studies was read in full and assessed against the inclusion criteria. Full text studies that did not meet the inclusion criteria were excluded and reasons for exclusion are included in a Preferred Reporting Items for Systematic Reviews and Meta-Analyses (PRISMA) flow diagram (see Figure 1) along with the results of the search. Any disagreements that arose between the reviewer pairs was resolved through discussion with another pair from within the review team.

Exclusion criteria:

1. Sample includes wider age range – unable to isolate data for older persons (e.g., mixed aged groups) or data for older persons is not able to be determined
2. Adults between 18 and 65 years or under 50 for indigenous populations
3. ‘Integrated care’ at the meso e.g. service/organisational level, macro/policy e.g. system level or lifespan level e.g. not specific to older people
4. No social component – clinical integrated care only
5. Quantitative data or mixed method papers with quantitative data as the primary data source
6. Language other than English
7. Publications other than peer-reviewed journal articles

### Quality appraisal

Eight members of the team worked in pairs to screen the full text of retrieved papers (n = 11) by the JBI Critical Appraisal Checklist for Qualitative Research [18] as shown in Appendix A Critical appraisal results of eligible studies. The checklist consists of ten questions and involved three distinct steps: filtering, technical appraisal, and theoretical appraisal (Hannes and Lockwood, 2011). Papers were included if both reviewers answered ‘yes’ to a minimum of seven of ten prompt questions, with disagreements resolved by consensus after reviewing the criteria. See Appendix A. Critical Appraisal results of eligible studies.

### Risk of bias (quality) assessment

The studies included in this systematic review were graded according to the JBI credibility criterion, which demonstrates the congruity between the research question and findings of the studies based on the theoretical frameworks used (JBI, 2014). Each of the included papers were assessed by two members of the research team and any disagreement between reviewers’ judgements were resolved by third members.

### Data synthesis

The meta-aggregation process is grounded in pragmatism and transcendental phenomenology [18]. This process requires the findings capture the whole phenomenon of interest to ensure the methodology used for the study is also embedded within these findings and retains the perspective or context provided by the study authors [20]. The findings from included papers, with illustrating quotes, constitute the first step of the meta-aggregation process. Findings are then aggregated into categories according to similarity in meaning. Further analysis of the categories shapes the synthesised statements. Following this process our statements incorporated key factors identified as clinical and social outcomes of integrated care for older people.

## Results

Characteristics of included studies are shown in Table 1. The majority of the included articles originated from Europe with remaining studies from Australia and Brazil. For this systematic review, eight qualitative papers [21,22,23,24,25,26,27,28], two mixed methods papers [29,30] and one paper using embedded case studies [31] met the inclusion criteria.

**Table 1:** Characteristics of included studies.


Title of study	The Coexistence center for elderly people and its importance in the support to the family and the Health Care Network	Social participation of people with cognitive problems and their caregivers: a feasibility evaluation of the Social Fitness Programme	A coordinated preventive care approach for healthy ageing in five European cities: a mixed methods study of process evaluation components	Driving forces for home-based reablement: A qualitative study of older adults’ experiences	Interprofessional working in hospice day care and the patients’ experience of the service	The over 75 Service: Continuity of Integrated Care for Older People in a United Kingdom Primary Care Setting	Encouraging older people to engage in resistance training: a multi-stakeholder perspective	Identifying ‘value’ in day care provision for older people.	Evaluation of a transition care cognitive assessment and management pilot	Experiences of Community-Living Older Adults Receiving Integrated Care based on the chronic care model: a qualitative study	Health and social care planning in collaboration in older persons’ homes: the perspectives of older persons, family members and professionals

Author/s (year)	Derhun FM, Scolari G A De Souza, Castro, VC, De Salci, MA, Baldissera, V DA & Carreira, L, 2019	Donkers, HW, Van Der Veen, DJ, Vernooij-Dassen, MJ, Nijhuis-Van Der Sanden, MWG & Graff, MJL., 2017	Franse et al. … 2019	Hjelle KM, Tuntland H, Førland O & Alvsvåg, H 2017	Lee, 2002	MacInnes J, Baldwin J & Billings J, 2020	Pettigrew et al., 2018	Powell & Roberts 2002	Renehan, E, Haralambous, B, Galvin P, Kotis M, & Dow B, 2014	Spoorenberg, SLW, Wynia K, Fokkens, AS, Slotman, K, Dremer HPH, Reijneveld, SA, 2015	Sundstrom et al. 2018

Country	Brazil	The Netherlands	United Kingdom, Greece, Croatia, the Netherlands and Spain	Norway	UK	UK	Australia	UK	Australia	Netherlands	Sweden

Aim of study	To know the perception of the elderly people’s family about the importance of a coexistence center (day centre focused on the socially vulnerable, dependent IADL but independent in basic ADL) on family support and on the Health Care Network (HCN).	Determine feasibility of a tailor-made intervention (the Social Fitness Programme), aimed at improving social participation of people with cognitive problems and their caregivers, in terms of acceptability, demand, implementation, practicability and limited efficacy	Evaluate specific process components of the Urban Health Centres Europe (UHCE) approach: a coordinated preventive care approach aimed at healthy ageing among older persons in community settings of five cities in the United Kingdom, Greece, Croatia, the Netherlands and Spain	Describe how older adults experience participation in reablement.	Enhance understanding of hospice day care through an in-depth qualitative case study to answer: 1 How does the interprofessional team work to provide care? 2 How is this experienced by patients and how do they spend their time?	Explore the concept of continuity of care in relation to integrated care, for frail, older people in the United Kingdom as part of the European SUSTAIN project	Investigate various stakeholders’ perceptions of how older people can be encouraged to commence and continue resistance training	Identify value in day care provision for older people 1. To identify the characteristics of the elderly populations receiving different types of day care and develop criteria for attendance. 2. To determine whether achievement of a negotiated goal(s) is the most appropriate outcome measure for elderly people	Evaluate the implementation and effectiveness of the TC CAMP. The evaluation sought to explore the perceptions of staff and family carers, and outcomes for the person with dementia.	Evaluate the opinions and experiences of community-living older adults with regard to integrated care and support, along with the extent to which it meets their health and social needs	Gain a deeper understanding of the HSCPC-meeting from the perspectives of older persons, family members, and professionals.

Methodology	Qualitative	Qualitative	Convergent mixed methods evaluation design (Creswell & Plano Clark, 2018) alongside the effect evaluation of the UHCE approach.	Qualitative arm of larger study on reablement in home-dwelling adults, including a small RCT. Recruitment based on referral to home-based services. Invited to participate in new	Framework of interprofessional working within qualitative paradigm	Multiple embedded case study design. Reported in another paper.	Qualitative	Qualitative	Mixed methods	Qualitative	Hermeneutic philosophy

Method	14 Semi-Structured Interviews undertaken by masters degree students, at a date and place of choosing by relatives. Interviews were recorded and transcribed and lasted an average of 20 minutes. Transcripts were thematically coded	Qualitative research methods (focus group discussions, interviews, collection of treatment records) and applied thematic analyses.	Quantitative data from a questionnaire and quantitative/qualitative data from log- books were collected among older persons involved in the approach. Qualitative data from focus groups were collected among older persons, informal caregivers and professionals involved in the approach.	Content analysis	Case study with interviews, observation, document analysis. The ‘case’ being a specialised hospice day care unit, including all patients & professionals within a 3-month period	Qualitative interviews with users & carers; demographic questionnaire for users & carers; interviews with professionals; focus group with managers & professionals delivering the service; documentary analysis of care plans; minutes of steering group meetings and researcher’s field notes.	A combination of interviews and focus groups was used to allow extensive discussions of relevant topics and the probing of new issues as they arose.	Mixed methods Interviews were conducted at time of entry to the service, following discharge or review three months later. Standard assessment instruments were used to collect data on functional status. Additional qual data was collected via interview.	File audits, focus groups and individual interviews with family/carers and staff, including nursing, management and allied health. Discharge destination service interviews were also conducted.	Semi-structured interviews were conducted with 23 older adults receiving integrated care and support	Interviews with older people (n=7), interviews with older persons and family (n=3), interviews with family member (n=5) and focus groups with professionals (n=10)

Quality measure	8	8	8	7	9	9	7	7	6	8	10

Phenomenon of interest	The coexistence center providing daytime stay of older people and care directed, mainly, to health promotion of older people as part of the Brazilian model of integrated care	Feasability evaluation based on experiences from professionals (programme deliverers), people with cognitive problems and their caregivers (programme recipients) of the tailor - made Programme. A cyclical process was applied. Treatment goals. Within the goal setting phase, priorities for the intervention were set.	Coordinated preventive care interventions on quality of life and independent functioning among older persons	Reablement - Perspectives of the older people themselves	Professional team working and patient experience of the service of Hospice Day Care	Integrated care viewed through the lens of the continuity of care hierarchy.	Role of resistance training in later life	The views and experiences of service users and professionals alongside information relating to costs.	Experiences of TC CAMP and perceptions of benefits and possible service gaps.	1) How do older adults experience the effects of aging? 2) How do older adults experience the care and support offered by a CCM-based integrated care model?	Understanding of value of HSCPC meeting from older people and HCPs perspective.

Setting	Community	Community	Community settings of five cities in the United Kingdom, Greece, Croatia, the Netherlands and Spain	Community-dwelling/at home	Hospice Day Care	Participants’ homes	Centres that offer resistance training programmes for older people in metropolitan and regional Western Australia	Three different day care settings: Day centre, outreach serv ice and day hospital	Transition Care Cognitive Assessment and Management Pilot (TC CAMP) funded via six restorative care places in a residential care facility	Embrace population-based integrated care model for community-living older adults.	Participants’ homes

Participants	14 relatives of older people attending the coexistence centre, coexistence center (day program) in a city in the interior of the state of Paraná, Brazil.	14 dyads of community dwelling people with cognitive problems and their caregivers (programme recipients) who wished to maintain or increase social participation	The target population consisted of persons living independently, aged 75 years or older, who were, according to their physician, able to participate in a care-pathway for at least 6 months	Eight older adults	Interprofessionals working in a hospice day care facility. The patients attending the hospice day care facility.	1) Users and carers: The inclusion criteria for users was 75 years of age or older, living at home, with multiple health and social care needs, in receipt of the service for a minimum of 12 weeks, and cognitively able to participate in the study. Informal caregivers of users were also invited to participate. 2) Managers and Professionals delivering the service. 3) Steering group consisting of managers and professionals was set up at the start of the SUSTAIN project.	Instructors (n=18) and centre mangers (n=24) were interviewed. Phase ii four focus groups with other relevant stakeholders (health practitioners n= 13 & older people n = 24).	day care attendees (n=45) (15 from each of the three settings), where applicable their informal carers and focus groups with members of the three teams.	Family carers of clients in TC CAMP (interviews). Staff including nursing, management and health service staff (interviews, focus groups) (pg 137). Clients were not interviewed as not able to give informed consent	23 older adults receiving integrated care and support	Ten older persons, eight family carers, 22 health care professionals

Data analysis	Content analysis technique in the thematic modality	All qualitative data (four focus groups and 13 interviews) were recorded and transcribed verbatim. These transcripts and 23 OT and PT treatment records were thematically analysed through a content analysis.	Quantitative data were analysed by means of descriptive statistics and multilevel logistic regression models. Qualitative data were analysed through thematic analysis.	Content analysis	Development of propositions following data coding and grouping, plus percentages of time participants spent in an activity.	Qualitative data was analysed thematically using Flick’s approach which involved bringing predetermined templates to the data, in this case the interview and focus group schedules. Quotes were sorted into categories and coded according to their origin. Each category was organised into themes using the quotes to justify interpretation. Quantitative demographic data was analysed using descriptive statistics.	Inductive approach to coding	Cost data was also collected from each of the three settings.	Qualitative data including perceptions of nursing staff, family/carers, discharge facilities and other key stakeholders were subject to content and thematic analysis	Data analysis was based on the grounded theory approach.	Hermeneutic analysis

Findings	From the family carer’s perspective, the older person’s participation in the coexistence center was an alternative to support care and institutionalisation, provided time for carer self-care and to maintain or engage in the formal work; time spent at the centre positively influenced the relationship from the family’s perspective toward the person. The performance of the coexistence center offered support to the family in the care of the elderly person.	Dyads formulated multiple intervention goals, ranging from a total of four to nineteen goals per dyad, with a range of 1 to 5 goals and a median of 2,5. In total, 34% of all goals (38 from a total of 111 goals) comprised increasing social participation. After prioritising, all dyads included at least one goal for social participation on level two on our operational model for social participation. The OT coordinated the interdisciplinary collaboration including sharing of information with the dyads’ GP, the PT and WP. The intervention was feasible according to stakeholders, and showed promising results. Feasibility and barriers. First, an acceptability barrier: discussing declined social participation was difficult, hindering recruitment. Second, a demand barrier:	Having limited function was associated with non-enrolment in falls and loneliness care-pathways (both p < .01). The mean rating of the approach was 8.3/10 (SD 1.9). Feeling supported by a care professional and meeting people were main benefits for older persons. Mistrust towards unfamiliar care providers, lack of confidence to engage in care activities and health constraints were main barriers towards engagement in care.	Themes: My willpower is needed; being with my stuff and my people; the home-trainers are essential; and training is physical exercises, not everyday activities. Intrinsic and extrinsic motivation influence reablement with some people needing more extrinsic motivational support after the time- limited reablement period is completed. The reablement team encouraged and supported the older adults to regain confidence in performing everyday activities as well as participating in the society. The municipal health and care services need	How the propositions fit into integrated care. Key forms of integrated care can include Integrated care within one sector (eg, within mental health services through multi-professional teams or networks) Integrated care between providers and patients to support shared decision-making and self-management.	Themes of continuity of care: International domain with subthemes 1) willingness to share information: Overall, there was a willingness to share information across organisations and amongst different professionals although the need to share information which was not perceived to be relevant to all agencies was questioned: “It’s kind of working together and just sharing information, rather than thinking ‘Oh we’re the district nurses and that’s the GP surgery’ and not sharing information. If we’re told something and we think it would be valuable for them to know, we’ll always pass that	Results indicate that the need for personalised attention in the establishment and maintenance phases of a resistance training programme can constitute both a positive and negative aspect of older people’s experiences. The negative aspects were identified as a series of tensions between the need for personalised attention and (a) the desire to participate in physical activity within social groups, (b) a preference for activity variation, (c) a dislike for large centres where personalised guidance is often available	Patient of the outreach service ‘valued opportunities for social contact alongside ‘exercises’ to regain their mobility. They appreciated discussion about realistic goal setting in terms of mobility improvement relevant to their home situation. There was general agreement that an attempt to negotiate goals with patients was implicit in their practice, although this was more evident in relation to particular situations where a formal plan had been agreed.	This evaluation found that it was possible to provide appropriate transition care to people with cognitive impairment who exhibited behavioural and psychological symptoms of dementia. The TC CAMP achieved length of stay and readmission rates that were comparable with transition care for cognitively intact people.	Responses of participants concerned two focus areas: 1) Experiences with aging, with the themes “Struggling with health,” “Increasing dependency,” “Decreasing social interaction,” “Loss of control,” and “Fears;” and 2) Experiences with Embrace, with the themes “Relationship with the case manager,” “Interactions,” and “Feeling in control, safe, and secure”.	Four themes emerged: 1.Unspoken agendas and unpreparedness(older people unclear about who was who in the HSCPC meeting, most older people came to the meeting unprepared as they were not sure what to expect or what to prepare. Overall, often ambiguity about the meetings.) 2.Security and enhanced understanding (older people appreciated the meetings and felt understood and meting at home meant the older person felt safe and was able to explain their home situation - which contributed to enhanced understanding of the older person. 3.Asymmetric relationships (older people did have some difficulty joining in conversation, felt they could not always speak for themselves or defend their interests.

		some people with cognitive problems lacked motivation to improve declined social participation, sometimes in contrast to their caregivers’ wishes. Third, implementation and practicability barriers: shared decision-making, focusing the intervention and interdisciplinary collaboration between healthcare providers.		to consider individualised follow-up programmes after the intensive reablement period to maintain the achieved skills to perform everyday activities and participate in society. The support must be adjusted to the older adults’ resources and health in their process of regaining confidence to perform activities themselves.		information over” (M/P6) From a user and carer perspective, information sharing was not always apparent to users and carers of the service who recognised that whilst information was shared amongst staff within the medical practice, this was not the case for outside agencies: 2) mechanisms for information sharing: Information was shared in monthly multidisciplinary team meetings.	yet the surroundings can be considered unappealing, (d) cost issues and (e) the need for flexibility in attendance.				Not always fully involved and unsure of some decisions made - may be insufficient time to establish symmetrical relations). 4.Ambiguity about the mission and need for follow-up: older people unsure about the need for HSCPC meeting.


**Figure 2:**
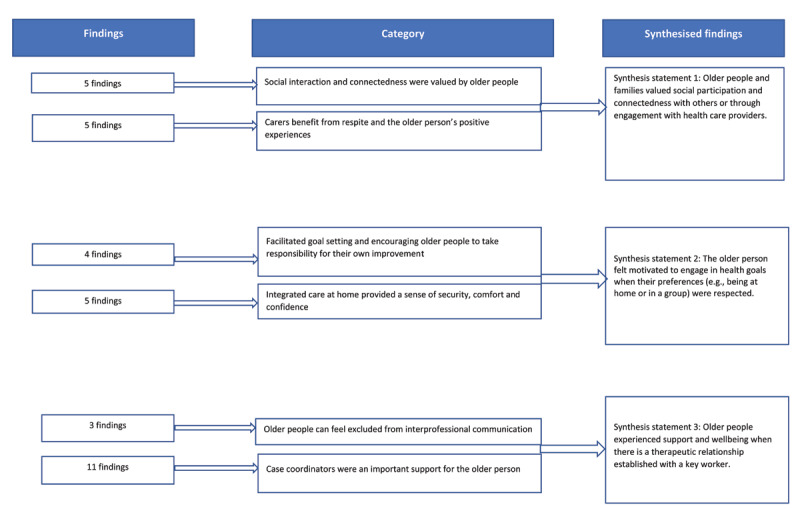
Meta-aggregation of findings.

From the 11 studies, 33 findings were extracted and allocated to six categories based on similarity of meaning (see Appendix B): older people valued social interaction and connectedness; family carers also benefitted from these positive experience as well as from periods of respite; goal setting and encouraging older people to take responsibility for their own improvement; the security, comfort and confidence provided by the home setting; older people can feel excluded from interprofessional communication; and case coordinators are an important source of support for the older person. These six categories were agreed amongst the team and aggregated into three synthesis statements: Older people and families valued social participation and connectedness with others or through engagement with health care providers; The older person felt motivated to engage in health goals when their preferences (e.g. being at home or in a group) were respected; Older people experienced support and wellbeing when there is a therapeutic relationship with a key worker (see Figure 2).

### Synthesis statement 1: Older people and families valued social participation and connectedness with others or through engagement with health care providers

Social participation and connectedness with others in group activities or through engagement with health care providers provided positive experiences for older people with connectedness being an outcome of integrated care. Two categories supported the first synthesis statement.

#### Category 1.1 Social interaction and connectedness

Four of six studies made references to social participation and connectedness [23,26,29]. The ability to meet and interact socially with others was commented on by both care recipients and their care givers as a key benefit of the integrated care programs beyond the benefits of the “actual exercise” [25]. Older people appreciated the opportunity for social contact with other attendees and the support they received from these relationships, some of which were new, others were longstanding. For example, the following quote is from a senior citizen and informal caregiver:

“Many of the women participating in these classes, we were already acquainted with from the past. I met some others during the classes. It is the human relationship, we helped each other.” (Senior citizen Pallini and informal caregiver) [29, supplementary file, table S3A].

Additional benefits of connectedness were derived from individual team members being cheerful and friendly and the companionship resulting from the social interactions appear to be key to the success of older people’s involvement in specific programs or interventions. The social aspect was identified as being the “biggest attraction” [25].

#### Category 1.2 Carers benefit from respite and the older person’s positive experiences

Two studies identified the benefit that carers received from the older person’s positive social experiences while being out of the home [21,22]. One study reported carers appreciated the older person not being alone, “without company” during the time spent at the coexistence centre and the older person being returned home to family at the end of the day [21]. Another positive outcome described by carers was increased engagement between the family and the older person: “After he began to participate in the coexistence centre, we have even more dialogue among the family. He has more fun […] He got much nicer” [21].

### Synthesis statement 2 – The older person felt motivated to engage in health goals when their preferences (e.g., being at home or in a group) were respected

When integrated care provided the opportunity to adapt to suit the older person’s preferences, the older person was motivated to participate. Nine findings from two categories supported this synthesis.

#### Category 2.1 Facilitating goal setting and encouraging older people to take responsibility for their own improvement

Four findings from three studies [23,26,29] supported this category in which the setting of realistic goals relevant to the older person’s home setting and where they were encouraged to feel confident in their performance helped older people to take responsibility for activities. Recovery was perceived to be “faster” because of the encouragement by the reablement team providing an implicit patient-reported indicator of a clinical outcome [23].

#### Category 2.2 Integrated care at home provided a sense of security, comfort, and confidence

This category was supported by five findings from three studies [23,28,29]. Receiving visits within the comfort of home was a key enabler for some older people, creating a sense of autonomy, confidence, and value. In the home setting, the older person felt safe and better understood by care providers [28]. Regaining confidence was a key theme in two studies [23,29] with the perception of the older person exhibiting a “more comfortable and open attitude” also indicating confidence from being on “home turf” [28]. Importantly, being connected to neighbours and the familiar environment of the home was also a contributing factor to the success of the integrated care program [23].

### Synthesis statement 3: Older people experienced support and wellbeing when there is a therapeutic relationship established with a key worker

Communication involving all stakeholders was integral to successful integrated care outcomes. When the older person was at the centre, communication led to empowerment, feeling in control, and facilitated desired outcomes. Having clear lines of communication with a central person or key worker also facilitated communication. Two categories with 14 findings supported this synthesis.

#### Category 3.1 Older people can feel excluded from interprofessional communication

Communication was seen to be important but sometimes difficult and depending on the strategies used to involve and/or motivate the older person, and the capabilities of the older person to interpret the information, the older person may not always feel involved or prepared in a team environment. “You’re sort of unprepared, because you sort of don’t know what they want” [28]. Older people also experienced being talked about as one person reported: “Yeah, they talked about all sorts of things, and mostly they talked ABOUT me. (…). She talked up a storm. And so, I responded to what she said. I can’t recall what she was asking about” [28].

#### Category 3.2 Case coordinators are an important support for the older person

This category was represented by 11 findings and several factors were identified to characterise successful outcomes. These included a continuous point of contact in the role of care co-ordinators or case managers who acted as a conduit for information: “The [case manager] is a real source of information for us. We regularly have questions about one thing or the other, and she tries to find answers for us. And she follows up on it too” [27].

The development of ongoing relationships with accessible and flexible care providers contributed to the success of a model or program. The role of the care coordinator/case manager provided the relational continuity which was felt to be key to building trusting and positive relationships when helping older people progress their care goals, as reported: “… she’s on a level with you rather than looking down at you, and that alone is worth a lot. And she talks like we do [in dialect], and she’s very down to earth. We say she’s a good one, and, as my husband says, we wouldn’t want to be without her” [27]. For carers too, this point of contact was invaluable: “I wouldn’t know who else to contact in the whole process … she [the CNC] was really, really good, she was fantastic – she couldn’t have done more for us … I highly recommend her services”[Relative 5]” [30]. This role established rapport and trust: “I trust them. You know, I mean this is the difference. The rapport is totally different with somebody that will listen to the patient than somebody that tells you what you’ve got to do” [31].

The accessibility and responsiveness of these professionals was highly valued: “You’ve only got to ring up the surgery and she’s here in about 3 minutes if you really need her. She’s always here if I badly need her” [31].

## Discussion

In examining the findings of the outcomes of integrated care at the individual level for older people, three synthesis statements were developed. These statements provide a rare insight into outcomes that are meaningful to older people, showing that the older person is motivated and engaged by integrated care initiatives that include social needs with more clinical care. Older people value integrated care when it incorporates care coordination, individual choice related to the context of care and goal setting driven by person-centred communication. These findings can inform services globally as they move to increase the intersection between health and social care services through redesign that better meets the needs of the older person.

Initially we had hoped to describe self-reported outcomes of integrated care for older people across multiple health domains; however, physical activity alone was identified [23,25].

### Older people and families valued social participation and connectedness with others or through engagement with health care providers

These synthesised findings reflect the value of social participation for older people who appreciate either the social aspect of a program or receiving home visits from key workers who are able to see the older person in their home context “as a person who needs support” [23]. Feeling connected, and the enjoyment that this brings, included having fun while involved in physical activity [25]. In the context of increasing and existential loneliness arising in many older people, especially the frail elderly [32], social interaction is a clear outcome of the integrated care activity [25]. Older persons valued meeting others [24,25], helping some to open up to others [29] as well as helping others to cope with physical impairments or illness [27].

Carers also benefitted from the older person’s positive experiences as they received much needed respite from care provision. Without, primarily, daughters on the frontline of care providing essential support to the older person and integration work, care would not meet the needs of the older person [33].

### The older person felt motivated to engage in health goals when their preferences (e.g., being at home or in a group) were respected

Integrated care goal setting motivated older people to work with health professionals [34]. Older people achieve reablement goals if coordinators apply enabling behaviours to empower older people to make “choices” that promote their own wellbeing goals [35].

Older people are more likely to pursue and achieve their goals, when they are involved in their setting as opposed to having these thrust upon them by the health care system [23,36]. In this way the older person is motivated to take part in a social or physical activity and to take control of this aspect of their care. Asking the older person what they would like to do is an obvious question but none of the papers provided insights into how individual goals were set.

As previously identified, goal plans related to integrated care focus on improving health-related problems and addressing client needs and priorities [34]. However, the older person’s complex needs encompass fear of increasing dependency, decreasing social interaction and a loss of control [27]. Social care interventions may best support or address these needs, although none were identified in this review. Instead, individualised care planning and involvement in decision making appeared to help the older person find purpose [23], which is a strong incentive in combatting the socially stigmatising constructs related to ageism, as well as achieving one’s own goals [37].

In our findings, engagement in physical activity and the location in which this took place was as important to the older people’s health outcomes as the social component. Being outdoors in the natural environment is a positive outcome for older people [23,25] and evidence shows that being in the outdoor, natural environment provides improvement in overall wellbeing, countering fatigue and poor-quality sleep [38]. The environment provides holistic benefits beyond the program goals of becoming physical fitter or being enabled to mobilise and this includes the value of care being provided in the home setting. A positive outcome of integrated care being provided in the home setting was demonstrated with participants feeling comfortable and secure in this environment [23,27] as a connection with one’s everyday life made the process more meaningful [23]. A setting that provides comfort, safety and control [39] is as important an enabler of health outcomes as other key enablers, such as a trusted care coordinator and good communication between the team [23].

### Older people experienced support and wellbeing when there is a therapeutic relationship with a key worker

The synthesised findings indicated that older people benefited from integrated care that provided clear communication and appropriate information, regular contact with a familiar trusted healthcare provider, individualised care planning and shared decision making [15].

This synthesis statement confirms the important outcomes of providing coordinated integrated care and the pivotal role care coordinators, and case managers have in the lives of older people. Our included studies show how these roles generate trust, provide support and even friendship, as well as enabling access to information, in some cases providing ‘an open line’ when problems arise [31]. This level of responsiveness to the needs of the older person encapsulates the relational, informational and organisational enabling elements of integrated care [15]. Moreover, there is strong link between case coordination and enabling health literacy, especially in the context of multiple chronic conditions. People aged over 65 years and with multiple chronic diseases have been found to experience greater health literacy difficulty than those under 65 years, particularly in engaging with health care professionals, using health services and finding health information [40,41]. Integrated care may bypass the problems caused by lack of coordination with older people and their caregivers becoming anxious and uncertain about their health care decisions [41].

The level of communication needed to ensure satisfactory service provision is reinforced by family carers who report the input of the interdisciplinary team being of utmost value [22]. Integrated care is able to bring together all stakeholders and reduce fragmentation and within this finding is the evidence that this is happening, albeit in a very limited way.

However, of note, is the inclusion of negative aspects experienced by older people including findings related to unspoken agendas, being talked about, miscommunication and information sharing which were issues identified by older people and health care professionals alike [28,29]. These highlight the challenges for older people related to multiple stakeholder engagement when they are not central to processes and experience disempowerment. When integrated care includes care coordination the outcome is older people feel more secure, well informed and, ultimately, motivated to keep informed and involved in their own care [15,42].

Consistent with earlier findings on the limited reported effects of integrated care on service users [17], there is little evidence that integrated care is working well from the older person’s perspective. Importantly, medication management, pain management, quality of life indicators and other vital clinical and social outcomes were not captured in the qualitative findings. There was some evidence of the older person’s lived experience regarding the benefits of integrated care and this has also been described elsewhere [15]. Patient reported outcome measures (PROMs) have an important role in patient-centred health care as these inform care provision. Older people may be encouraged to discuss the physical, psychosocial and non-medical issues leading to improvements in the therapeutic relationship between clinicians and themselves [43]. However, Briggs and colleagues (2018) [44] reported less emphasis on patients’ experiences of care with more interventions focused on service provision. This highlights a gap in the reporting of outcomes at the individual level e.g PROMs and Patient-reported experiences of care (PREMs) which is necessary for quality care that is informed by the older person receiving the care.

Overall, our findings support previous literature that the voice of the older person is not well represented in the research on integrated care [17,27]. Further, the voice of the growing number of older people above the age of 85 years was especially under-represented. Beyond the community context within which aged care services are provided, residential aged care consumers were also absent from the studies pointing to a lack of opportunity to implement and integrate care in this setting where our oldest old have the most complex health and social care needs, including social isolation, loneliness and high level dependency [32,45].

## Strengths and Limitations

This is the first paper that specifically looks at the clinical and social outcomes of integrated care outcomes for older people. While there is more research at the micro-system level, the focus of this search at the individual level is extremely important to gauge impacts for the older person, to determine what works best and what quality improvements are necessary. The search did not yield many studies from which to draw out the outcomes under review. We suspect that further studies may be buried within the reablement literature, or literature on person-centred care. What we have captured here is a limited body of research on what the older person, their families and carers have expressed in this field. The limited focus on outcomes for individuals arising from integrated care initiatives makes it difficult to draw strong conclusions about impact.

Further, some of the papers are quite dated (e.g., Lee 2002) which is surprising and may point to a wider definitional problem that has overlooked research in the field.

A more obvious limitation is the lack of research identifying and discussing social care outcomes of integrated care for older people. Social aspects of included studies represented more of an add on to the health services or physical activity program and were not truly holistic in aim.

## Conclusion

This review found outcomes of integrated care for older people are situated in the positive social interactions within groups or with key workers. The high level of support, information and enabling of the older person through these roles motivated older people to engage in their health goals. Given the importance of these roles to older people, embedding these roles across services, including residential aged care, are recommended.

The limited qualitative data also warrants further research into the clinical and social outcomes of integrated care for older people.

## Additional File

The additional file for this article can be found as follows:

10.5334/ijic.6469.s1 Supplementary data associated with this article can be found in Appendices A and B.
