# Supplementary material for: What are the Clinical and Social Outcomes of Integrated Care for Older People? A Qualitative Systematic Review
Source: Int J Integr Care. 2022 Sep 7;22(3):14. doi: 10.5334/ijic.6469 (PMC9504020; doi:10.5334/ijic.6469)
Supplement: Supplementary file 1 — Supplementary data associated with this article can be found in Appendices A and B. [file ijic-22-3-6469-s1.zip › s1-ijic-6469_karacsony/6469-26448-1-SP.docx]

Appendix A. Critical appraisal results of eligible studies

| **Study** | **Q1** | **Q2** | **Q3** | **Q4** | **Q5** | **Q6** | **Q7** | **Q8** | **Q9** | **Q10** |
| --- | --- | --- | --- | --- | --- | --- | --- | --- | --- | --- |
| (Derhun et al., 2019) | N | Y | Y | Y | Y | Y | N | Y | Y | Y |
| (Donkers, van der Veen, Vernooij-Dassen, Nijhuis-van der Sanden, & Graff, 2017) | NA | Y | Y | Y | Y | Y | N | Y | Y | Y |
| (Franse et al., 2019) | NA | Y | Y | Y | Y | Y | N | Y | Y | Y |
| (Hjelle, Tuntland, Førland, & Alvsvåg, 2017) | U | Y | Y | Y | Y | N | N | Y | Y | Y |
| (Lee, 2002) | Y | Y | Y | Y | Y | Y | Y | Y | U | Y |
| (MacInnes, Baldwin, & Billings, 2020) | Y | Y | Y | Y | Y | Y | N | U | Y | Y |
| (Pettigrew et al., 2019) | U | Y | Y | Y | Y | N | U | Y | Y | Y |
| (Powell & Roberts, 2002) | U | Y | Y | Y | U | N | N | U | N | Y |
| (Renehan, Haralambous, Galvin, Kotis, & Dow, 2013) | NA | U | Y | Y | Y | N | U | Y | Y | Y |
| (Spoorenberg et al., 2015) | Y | Y | Y | Y | Y | U | Y | Y | NA | Y |
| (Sundström, Petersson, Rämgård, Varland, & Blomqvist, 2018) | Y | Y | Y | Y | Y | Y | Y | Y | Y | Y |

  1. Is there congruity between the stated philosophical perspective and the research methodology? 
2. Is there congruity between the research methodology and the research question or objectives? 
3. Is there congruity between the research methodology and the methods used to collect data? 
4. Is there congruity between the research methodology and the representation and analysis of data? 
5. Is there congruity between the research methodology and the interpretation of results? 
6. Is there a statement locating the researcher culturally or theoretically?  
7. Is the influence of the researcher on the research, and vice- versa, addressed? 
8. Are participants, and their voices, adequately represented?  
9. Is the research ethical according to current criteria or, for recent studies, and is there evidence of ethical approval by an appropriate body?  
10. Do the conclusions drawn in the research report flow from the analysis, or interpretation, of the data?

(Lockwood et al., 2017)

Lockwood, C., Porrit, K., Munn, Z., Rittenmeyer, L., Salmond, S., Bjerrum, M., . . . Stannard, D. (2017). Systematic reviews of qualitative evidence. In A. E & M. Z (Eds.), *JBI Manual for Evidence Synthesis* (pp. 22-70). The Joanna Briggs Institute: JBI.
