# Supplementary material for: What are the Clinical and Social Outcomes of Integrated Care for Older People? A Qualitative Systematic Review
Source: Int J Integr Care. 2022 Sep 7;22(3):14. doi: 10.5334/ijic.6469 (PMC9504020; doi:10.5334/ijic.6469)
Supplement: Supplementary file 1 — Supplementary data associated with this article can be found in Appendices A and B. [file ijic-22-3-6469-s1.zip › s1-ijic-6469_karacsony/6469-26449-1-SP.docx]

Appendix B Synthesis summary

| Author/Study | Finding | Illustration |
| --- | --- | --- |
| **Category 1: Social interaction and connectedness** | | |
| Powell, J. Roberts, H. 2002. | Older people attending day care viewed the primary goal to be social interaction. | The immediate **warmth and friendliness** of staff was appreciated by those initially reluctant to attend. Help with personal care and the opportunity to consider other forms of support were valued. Carer relief, frequently experienced as “peace of mind” or an opportunity to “get out” was also highly (Franse et al., 2019)valued. (p. 162) |
| Hjelle, K. M.  Tuntland, H.  Førland, O.  Alvsvåg, H.  2017 | Being with my stuff and my people included dimensions of being known, having regular home visits and having the opportunity to continue taking part in leisure activities and social life | I have lived here for a long time. Here I have all my good neighbours and it is important that they, friends and my family can visit me. It is important for me to go outside the home to have fresh air and meet some people. I enjoy myself here in my home. You feel at home because you have stuff and people all around you. I think it has been a great deal that the reablement service came home to me. [participant no. 8] (p 1585) |
| Hjelle, K. M.  Tuntland, H.  Førland, O.  Alvsvåg, H.  2017 | The essential support of the reablement team as a co-partner was expressed in various ways: ‘they encourage me’, and ‘someone who provides care and sees me as a person who needs support’. | “The reablement team was so nice people, they were a bright spot, and very cheerful and friendly. They came several times a day in the beginning of the reablement period. I was taken care of. I knew they were coming, and I believe it is very positive, that you in a way recover faster. [participant no. 8] (p. 1586) |
| Franse CB, Zhang X, van Grieken A, et al.  2019 | Older people valued meeting people | “Many of the women participating in these classes, we were already acquainted with from the past. I met some others during the classes. It is the human relationship, we helped each other.” (Senior citizen Pallini and informal caregiver)  “Yes, we laughed together. If you can laugh together with those people, you can do everything else, too.” (Senior citizen Rijeka)  “When you have just lost your husband you are very lonely so going to that and I go to a reading club at [charity organization] on Friday meet more people like myself who have lost husbands and wives.” (Senior citizen Manchester) p. 3697  “I liked it a lot, it helped me to open up to people.” (Senior citizen Valencia) p. 3696  They actually meet people and then they go off and have coffees and things like that together.” (Male, Centre Manager, Metropolitan area) p1814  (supplementary file Table S3A) |
| Pettigrew, S.  Burton, E.  Farrier, K.  Et al.  2018 | Older people enjoyed the social aspect. | “It seems to me, social is the biggest attraction. Whatever programme you want to introduce, social is the biggest attraction.” (Female, Seniors FG) p1813  “So there’s still the social aspect of it as well, because that, apart from the benefits of the actual exercise, that’s probably the biggest benefit for a lot of them. |
| **Category 2 Carers benefit from respite and the older person’s positive experiences** | | |
| Derhun, F.  Scolari, M.  Aparecida De Souza, G.  et al. 2019 | Carers experience time for themselves | ‘It is the only time I have, it is the time he is in the coexistence center. It is the time that I have to rest ..because it is not the whole day that he has to spend with me (F-6, p.4). |
| Derhun, F.  Scolari, M.  Aparecida De Souza, G.  et al. 2019 | Families experienced a reduction in stress and positive outcomes from the care service | ‘My mother was very much at home doing nothing….I was getting annoyed …but I was at my limit’ (F2, p.4) |
| Derhun, F.  Scolari, M.  Aparecida De Souza, G.  et al. 2019 | Older person responded positively to the service making care at home a more positive experience | ‘After he began to participate in the coexistence center, we have even more dialogue among the family. He has more fun […] He got much nicer’ (f-8, p.4). |
| Derhun, F.  Scolari, M.  Aparecida De Souza, G.  et al. 2019 | Older people have an alternative to institutionalised care | “My family said that when I could no longer take care of my uncle I should put him in a nursing home. But while he is here (coexistence center) we can handle [...]. In the nursing home, the contact with the family is lost and it is bad for him and for us. Here he comes in the morning and comes back in the afternoon and is not alone, without a company (F-5, p.3)”. |
| Donkers, H. W.  Van Der Veen, D.  Vernooij-Dassen, M. J.  Nijhuis-Van Der Sanden, M. W. G.  Graff, M. J. L  2017. | Older people with cognitive problems lacked motivation to improve their social participation | Caregiver ‘She does not feel like doing anything, but that is part of the disease I understand’ (i6, p.e57).  “As long as she stops sitting on the couch all day I will be satisfied, no matter what it takes”. (i6, p.e56) |
| **Category 3: Facilitating goal setting and encouraging older people to take responsibility for their own improvement** | | |
| Powell, J. Roberts, H. 2002. | Older people attending the Outreach Service desired realistic goals including improvement in mobility as the primary goal | Some patients had hoped for more improvement, they appreciated some discussion about the setting of realistic goals and making them relevant to their home situation. |
| Hjelle, K. M.  Tuntland, H.  Førland, O.  Alvsvåg, H.  2017 | Older people were encouraged to take responsibility in daily training | “I have the responsibility to train to get better. There is no one else who can do it. The team supervised me, and I asked questions in order to do it the right way, to have benefit of training activities of daily living (ADL). It wouldn’t be the same if the therapist did the activities for me, because then I had not remembered it, and I had not learned how to perform them.” [participant no. 8] (p. 1586)  “I didn’t train showering . . . I have showered all my life that is no exercises or training for me. It was one of my goals to manage showering, and I adapted the showering by having the towel and clothes just next. I cannot imagine me saying training showering. Another goal for me was to clean the floor. I practised or tried to clean the floor with the reablement team, I did not train to clean the floor. [participant no.7] (p. 1587) |
| Hjelle, K. M.  Tuntland, H.  Førland, O.  Alvsvåg, H.  2017 | Older people were encouraged to feel confident doing everyday activities on one’s own | “That is essential when I want to exercise and practice on my own. I have received much praise from the team. I believe it is because I have been active myself that I have recovered quite well now”. [participant no. 8] (p. 1586) |
| Franse CB, Zhang X, van Grieken A, et al.  2019 | Older people were motivated to take action | “Oh yeah! I changed my bed and bought another bed, a smaller one from [town in UK]; it is a bit higher than my original bed so when I get to park myself on it to get in it is ever so much better.” (Senior citizen Manchester)  “It was informative and it makes you think about a lot of things, they motivate you in certain things.” (Senior citizen Rotterdam)  (supplementary file Table S3A) |
| **Category 4: Integrated care at home provided a sense of security, comfort and confidence** | | |
| Sundström, M.  Petersson, P.  Rämgård, M.  Varland, L.  Blomqvist, K.  2018 | Older people appreciated the meetings and felt understood and meeting at home meant the older person felt safe and was able to explain their home situation. | Older persons: ‘Well, it was more comfortable to be at home and have it [the meeting] here ... I do not know, you feel so at home here.’ (Older Person, Interview 6) p. 151 |
| Sundström, M.  Petersson, P.  Rämgård, M.  Varland, L.  Blomqvist, K.  2018 | Meeting in the home with HCP provided a better understanding of the older person and how health problems were intertwined with the home environment.  This contributed to a better understanding of the older person. | Family members: ‘I can see another advantage of it… that you're just here at home… it makes for a more open attitude, a more comfortable and open attitude in some ways, more comfortable in that respect … like he can be a little bit more open to it on his home turf” p. 151 |
| Sundström, M.  Petersson, P.  Rämgård, M.  Varland, L.  Blomqvist, K.  2018 | Security and enhanced  understanding – almost all older persons appreciated having the HSPC-**meeting at home. They felt understood** and experienced it as satisfying to talk about what was troubling them and **felt comfortable about meeting at home.** | Professionals: ‘And then she talked a lot about her walker, it was important for her to go out with the dogs and so, so I knew she used it a lot (...) So I looked while I was there, I looked when I went out, and I saw that it was really not so usable anymore, but we changed after that.’ (Professional, Interview 8) (Fig 1, page 151) |
| Hjelle, K. M.  Tuntland, H.  Førland, O.  Alvsvåg, H.  2017 | Older people regained confidence through reablement at home | “In the early stage of the reablement, the professionals walked next to me up the stairs, I did not trust myself. I regained some confidence after intensive training, and they walked behind me. When the reablement was nearly coming to an end, I walked up and down the stairs myself, however, the staff was in the house. I felt completely secure when I knew they were in the house”. [participant no. 9] (p. 1586) |
| Franse CB, Zhang X, van Grieken A, et al.  2019 | Older people developed confidence | “Know it gives you confidence if people tell you everything is okay, you know like your bathroom and safe in front room, I think that is the most important.” (Senior citizen Manchester) (supplementary file Table S3A) |
| **Category 5: Older people can feel excluded from interprofessional communication** | | |
| Sundström, M.  Petersson, P.  Rämgård, M.  Varland, L.  Blomqvist, K.  2018 | Older people had difficulty with conversations. | ‘Yeah, they talked about all sorts of things, and mostly they talked ABOUT me. (…). She talked up a storm. And so I responded to what she said. I can't recall what she was asking about.’ (Older person, Interview 10) |
| Sundström, M.  Petersson, P.  Rämgård, M.  Varland, L.  Blomqvist, K.  2018 | Ambiguity about the mission and need for follow up. Older people felt unsure about the meeting. | ‘I don´t really know why [the meeting was held]. I think they…you know they arrange such meetings now and then.’ (Older person, Interview 9) |
| Franse CB, Zhang X, van Grieken A, et al.  2019 | Older people were unsure about written communications and difficult questions | “In some cases, they are truly hair-splitting issues and often you do not know what to fill in. Yes, you have to be educated in order to determine these levels.” (Senior citizen Rijeka) |
| **Category 6: Case coordinators are an important support for the older person** | | |
| Spoorenberg, S.L.W.  Wynia, K.  Fokkens, A.S. et al.  2015 | Older people developed a relationship with the case manager | “I think she’s a friendly woman, and she’s on a level with you rather than looking down at you, and that alone is worth a lot. And she talks like we do [in dialect], and she’s very down to earth. We say she’s a good one, and, as my husband says, we wouldn’t want to be without her.” (C1F) p.11 |
| Spoorenberg, S.L.W.  Wynia, K.  Fokkens, A.S. et al.  2015 | Older people confided in their case managers | “I don’t tell my children everything either. In that respect, I’m quite closed. But I’ve taken her [the case manager] into my confidence and I tell her everything. Then you’ve got someone you can tell it to, haven’t you? And it doesn’t go any further.” (C5M) p. 11 |
| Spoorenberg, S.L.W.  Wynia, K.  Fokkens, A.S. et al.  2015 | Older people felt emotionally supported by the case manager | “It’s as if you’ve got some support [. . .] I don’t want to put her [the case manager] on a pedestal, but she’s a real pillar of strength for us.” (C1F); “As far as empathy is concerned, she’s fantastic. **And the emotional support that she gives**. . . Her words are such a help. ‘We’ll never, ever turn our backs on you,’ she says.” (C2M) p.11 |
| Spoorenberg, S.L.W.  Wynia, K.  Fokkens, A.S. et al.  2015 | Older people felt that they were being monitored by their case coordinator | “Anything we tell her she brings up again the next time. [. . .] Without being prompted, but she’s aware of it. [. . .] And it’s the small things, but she takes good note of them.” (C1M) p.11  “Yes, she then says ‘I’ve spoken to the doctor and he thought this or he thought that’. Yes, we’re being looked after, I do have that feeling.” (C2M) p. 11 |
| Spoorenberg, S.L.W.  Wynia, K.  Fokkens, A.S. et al.  2015 | Older people were kept informed by their case coordinator | “The [case manager] is a real source of information for us. We regularly have questions about one thing or the other, and she tries to find answers for us. And she follows up on it too.” (C1F) p.12 |
| Spoorenberg, S.L.W.  Wynia, K.  Fokkens, A.S. et al.  2015 | Older people were encouraged by their case coordinator | “For example, she [the case manager] brought me a leaflet. Because there are computer lessons for seniors here in Stadskanaal, ‘And that’s just what you need,’ she said.” (C2M) p.12 |
| Spoorenberg, S.L.W.  Wynia, K.  Fokkens, A.S. et al.  2015 | Older people felt in control and safe knowing that the case manager was available | “I find it a great reassurance that she [case manager] says ‘We’re here if you need us.”; “If there’s anything I don’t know, I always talk about it with her.” (F12M)  p. 12 |
| Franse CB, Zhang X, van Grieken A, et al.  2019 | Older people valued the feeling that someone looked out for them | “[The Geriatric nurse practitioner] said that if something is wrong you can always call me. That was really nice.” (Senior citizen Rotterdam)  “Well I feel there is someone there looking out for you, you know, you are not ignored, they are out there wanting to help and, you know, it’s great.” (Senior citizen and informal caregiver Manchester) |
| Macinnes, J.  Baldwin, J.  Billings, J.  2020 | An identified care coordinator provides older people with easy access to services | “I think she [practice matron] is specified as my care co-ordinator so I do feel I’ve got an open line to her” (U3) |
| Macinnes, J.  Baldwin, J.  Billings, J.  2020 | Older people develop relational continuity | “ The rapport is totally different with somebody that will listen to the patient than somebody that tells you what you’ve got to do”. (U7, p.5). |
| Macinnes, J.  Baldwin, J.  Billings, J.  2020 | Older people value access to a professional | “You’ve only got to ring up the surgery and she’s here in about 3 minutes if you really need her. She’s always here if I badly need her” (U7, p.5) |

Franse, C. B., Zhang, X., Grieken, A., Rietjens, J., Alhambra‐Borrás, T., Durá, E., . . . Raat, H. (2019). A coordinated preventive care approach for healthy ageing in five European cities: A mixed methods study of process evaluation components. *Journal of Advanced Nursing, 75*(12), 3689-3701. doi:10.1111/jan.14181
